# A Dilated Convolutional Neural Network for Cross-Layers of Contextual Information for Congested Crowd Counting

**DOI:** 10.3390/s24061816

**Published:** 2024-03-12

**Authors:** Zhiqiang Zhao, Peihong Ma, Meng Jia, Xiaofan Wang, Xinhong Hei

**Affiliations:** 1 The School of Computer Science and Engineering, Xi’an University of Technology, Xi’an 710048, China; 2201221121@stu.xaut.edu.cn (P.M.); mengjia@xaut.edu.cn (M.J.); wangxfok@xaut.edu.cn (X.W.); xinhonghei@xaut.edu.cn (X.H.); 2 The Shaanxi Key Laboratory of Network Computing and Security Technology, Xi’an 710048, China

**Keywords:** crowd counting, dilated contextual module, contextual information, cross-level connection, dilated convolutional neural network

## Abstract

Crowd counting is an important task that serves as a preprocessing step in many applications. Despite obvious improvement reported by various convolutional-neural-network-based approaches, they only focus on the role of deep feature maps while neglecting the importance of shallow features for crowd counting. In order to surmount this issue, a dilated convolutional-neural-network-based cross-level contextual information extraction network is proposed in this work, which is abbreviated as CL-DCNN. Specifically, a dilated contextual module (DCM) is constructed by importing cross-level connection between different feature maps. It can effectively integrate contextual information while conserving the local details of crowd scenes. Extensive experiments show that the proposed approach outperforms state-of-the-art approaches using five public datasets, i.e., ShanghaiTech part A, ShanghaiTech part B, Mall, UCF_CC_50 and UCF-QNRF, achieving MAE 52.6, 8.1, 1.55, 181.8, and 96.4, respectively.

## 1. Introduction

**Figure 1:**
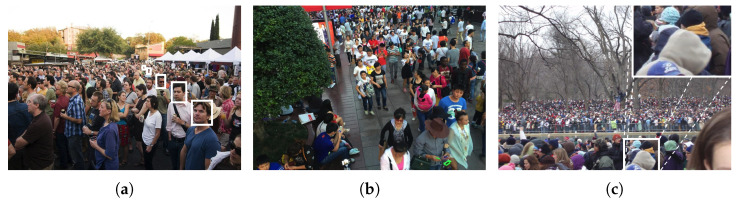
Challenges in crowd counting tasks (**a**) for perspective distortion, (**b**) for background noise, and (**c**) for occlusion.

The escalating urban residential population and the consequent rise in crowd density have significantly heightened the pressure on public safety in recent decades, evident in incidents such as stampedes. A pivotal challenge lies in accurately assessing crowd size in images, crucial for applications spanning video surveillance, traffic monitoring, pedestrian anomaly detection, and traffic control [1]. Crowd counting, fundamental in crowd analysis, underpins higher-level tasks. However, this analysis is confronted with several hurdles. Firstly, perspective distortion arises from varied distances of individuals from monitoring equipment, leading to differing head sizes across the crowd image (as depicted in Figure 1a). This distortion compromises counting accuracy and complicates individual detection. Secondly, complex real backgrounds can impede crowd detection; elements such as bushes (illustrated in Figure 1b) might be misidentified as crowds, adding to the challenge. Lastly, occlusion phenomena are prevalent in densely populated crowds (as seen in Figure 1c), presenting a unique obstacle to accurately identifying and enumerating individuals. These issues collectively contribute to the formidable task of crowd counting.

In recent years, the rapid evolution of deep learning technologies has opened up exciting new avenues for tackling the challenges inherent in crowd counting. Among these methods, convolutional neural networks (CNNs), serving as foundational models in deep learning, offer compelling advantages, such as automatic feature extraction and reduced manual intervention in end-to-end learning processes. Specifically tailored to address scale changes and occlusion stemming from perspective distortion, a range of approaches has emerged. These include methods based on multicolumn CNN architectures [2,3,4] and attention mechanisms [5,6], aimed at leveraging rich information for more precise crowd counting. Additionally, the integration of a scale regression module (SRM) [7] into lightweight convolutional networks has enabled efficient extraction of crowd features, effectively reducing network parameters and computational costs. Rather than relying solely on predictions from a single feature level, the utilization of a scale-adaptive selection network [8] has proven beneficial. This approach facilitates the learning of internal correspondences between scales and feature levels, thereby enhancing estimation accuracy. Furthermore, dilated convolution-based techniques [9,10,11] have gained prominence for capturing contextual information across deep scales, leveraging their larger receptive fields without inflating parameter counts. These advancements collectively represent significant progress in overcoming the complexities inherent in crowd counting tasks.

While CNN-based methods have demonstrated remarkable success in crowd counting, many of these approaches predominantly emphasize the utilization of deep feature maps, often overlooking the significance of shallow features in achieving precise counting. To tackle this limitation, we propose a dilated convolution-based CNN tailored for congested crowd counting. This novel architecture, named Cross-Level Dilated CNN (CL-DCNN), integrates contextual information from both shallow and deep features through cross-layer connections, thereby enhancing counting performance. Specifically, our approach introduces a Dilated Contextual Module (DCM) to address perspective distortion issues. The DCM comprises two branches: one dedicated to reducing channel count while preserving contextual information, and the other leveraging dilated convolutions to capture horizontal features across various scales. The outputs of these branches are concatenated to fuse deep and shallow features effectively. Within the CL-DCNN framework, multiple DCMs are cascaded in the CNN backend to capture complementary scale information. In the final stages, a fully connected layer incorporating 1x1 convolutions is employed to reduce the channel count to 1. This process generates the predicted density map, which is subsequently upsampled to match the original image size. This strategy facilitates direct loss calculation using the real density map, thus mitigating counting errors induced by downsampling. Additionally, CL-DCNN utilizes the entire image for training instead of relying on image patches, thereby enhancing estimation accuracy.

The primary contributions of this study are outlined as follows:

- Initially, by amalgamating several convolutional and dilated convolutional blocks into two branches, we introduce a dilated contextual module designed to address the issue of perspective distortion inherent in crowd images.
- Subsequently, we propose a novel dilated convolutional network aimed at fully leveraging the cross-layer connections across various feature maps. This network facilitates the fusion of contextual information while preserving intricate details within crowd images.

To assess the efficacy of the proposed CL-DCNN, we evaluate its performance across five publicly available crowd counting datasets: ShanghaiTech parts A and B, Mall, UCF_CC_50, and UCF-QNRF. Comparative analysis against state-of-the-art methodologies reveals that the CL-DCNN yields significantly lower estimation errors in crowd counting.

The subsequent sections of this paper are structured as follows. In Section 2, we present an overview of related works to contextualize the proposed approach. Section 3 offers a comprehensive exposition of the proposed CL-DCNN, encompassing detailed discussions on the Dilated Contextual Module (DCM) and the associated loss function. In Section 4, we undertake extensive experiments across five publicly available datasets, conducting comparative analyses with leading state-of-the-art methodologies. Finally, Section 5 presents the concluding remarks, summarizing the key findings and contributions of this study.

## 2. Related Works

In this section, we provide a concise overview of CNN-based approaches to crowd counting, encompassing both multicolumn CNN-based methods and dilated convolution-based methods. These approaches are delineated in the subsequent subsections for clarity and ease of reference.

### 2.1. Multicolumn CNN-Based Methods

Due to the variability in distances between monitoring equipment and individuals, perspective distortion can lead to significant scale variations in head images across different positions. Addressing this challenge, early research efforts have extensively employed multicolumn network structures. For instance, the multicolumn convolutional neural network (MCNN) [2] is introduced, comprising three convolutional layers with distinct kernel sizes and a feature fusing layer employing a 1 × 1 convolution. The MCNN’s columnar architecture enables the detection of heads of varying sizes within crowd images by accommodating different receptive fields. Similarly, the CountForest model [4], adopting a multicolumn structure, integrates a multi-branch convolutional neural network with deep regression forest techniques for crowd counting. By replacing the softmax layer with probabilistic decision mechanisms, CountForest effectively establishes mappings between image features and crowd counts in an end-to-end fashion. Furthermore, to adaptively encode contextual information across different scales, the CA-Net [3] is introduced as a trainable deep architecture. Through learning the significance of features at various locations within crowd images, CA-Net enhances the contextual understanding of the scene.

Furthermore, by integrating the U-net architecture with an Adaptive Scenario Discovery (ASD) module, Hafeezallah et al. proposed the U-ASD-Net [12] for crowd counting. Specifically, the U-ASD-Net employs a max-unpooling layer to upsample feature maps based on maximum locations, thereby replacing the nearest upsampling method in the U-part. The ASD component comprises three lightweight pathways, with two dedicated to capturing different crowd densities, each with distinct receptive fields. Additionally, the third pathway is designed to identify and model complex scenarios by recalculating pathway-wise responses. Incorporating scale information, the Scale-aware Representation Network (SRNet) [13] is introduced for crowd density estimation within an encoder-decoder framework. SRNet comprises two modules: the Scale-aware Feature Learning Module (SAM) and the Pixel-aware Upsampling Module (PAM). SAM adeptly models multi-scale crowd features at each level, employing varying receptive field sizes. Conversely, PAM enhances spatial resolution and enriches pixel-level semantic information, consequently bolstering overall counting accuracy.

Multicolumn approaches, while effective in certain contexts, are not without their limitations. Their reliance on multiple streams of convolutional layers to process the same image can lead to heightened computational costs and memory usage. Moreover, they may fall short in effectively capturing contextual information, a vital aspect for precise crowd counting. Furthermore, models employing multiple networks often boast numerous parameters and necessitate extensive calculations, posing challenges in achieving real-time crowd counting capabilities.

### 2.2. Dilated Convolution-Based Methods

Due to its capacity to acquire a larger receptive field without escalating the parameter count in deep learning models, dilated convolution has become prevalent in the literature on crowd counting for capturing contextual information at deeper scales. An early example is the CSRNet [9], which employs dilated convolution. This method comprises a front-end network and a back-end network. The front-end segment utilizes a pre-trained VGG16 network devoid of fully connected layers to extract image features. Conversely, the back-end network employs an extended dilated convolutional layer to broaden the sensing domain while preserving resolution, thus facilitating the generation of high-quality crowd density maps.

Subsequently, several variants of dilated convolution-based models have emerged for crowd counting, each offering advancements in succession. To address the scale variation stemming from perspective distortion, the Atrous Convolutions Spatial Pyramid Network (ACSPNet) [10] is introduced. This model conducts crowd counts and generates density maps for both sparse and congested scenarios. By employing atrous convolution within a spatial pyramid pooling module, it effectively expands the perception field of feature maps while preserving resolution. Zhu et al. [14] presented a crowd counting method termed the Dilated-Transposed Fully Convolutional Neural Network (DT-CNN), comprising a Feature Extraction Module (FEM) and a Feature Recovery Module (FRM). This architecture is tailored to produce high-quality density maps and accurately estimate passenger counts in densely populated metro scenes. Additionally, to tackle the issue of dramatic scale variations, the Perspective-Guided Fractional-Dilation Network (PFDNet) [11] was proposed for crowd counting. This model adeptly models continuous scale variation and selects appropriate fractional dilated kernels to adapt to different spatial locations.

These approaches have showcased the efficacy of dilated convolution in crowd counting, yielding superior performance compared to multicolumn-based methods. However, as the network depth increases, there is a risk of losing more detailed information. Given that crowd counting entails pixel-level regression, preserving intricate details, particularly in congested scenes, is paramount. Consequently, relying solely on dilated convolutions to expand the receptive field may lead to significant loss of detail in shallow features, potentially compromising crowd counting accuracy. To mitigate these concerns, we propose a Dilated Convolutional Neural Network-based Cross-Level Contextual Information Extraction Network (CL-DCNN) for crowd counting. This novel framework aims to preserve detailed information within shallow features while effectively leveraging cross-level contextual information.

## 3. Proposed Method

**Figure 2:**
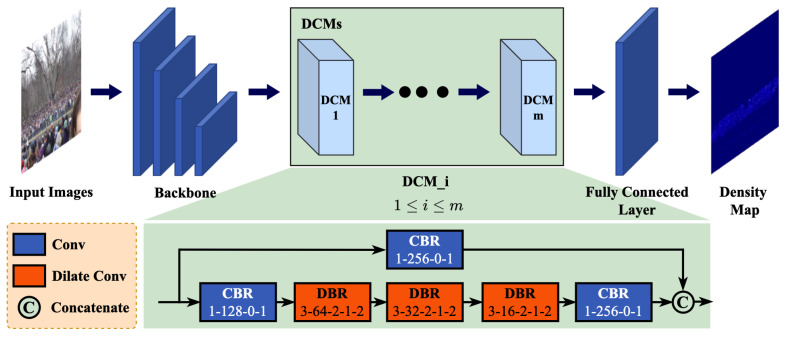
Network structure of the proposed CL-DCNN.

In our research, we introduce a novel approach, the Cross-Level Dilated Convolutional Neural Network (CL-DCNN), tailored specifically for crowd counting tasks. As depicted in Figure 2, the network comprises three key components: the backbone module, Dilated Contextual Modules (DCMs), and a fully connected layer. To enable the network to process input of varying sizes, we adapt the VGG16 architecture by replacing its fully connected layer with a fully convolutional layer. In Figure 2, we retain the first 10 convolutional layers of VGG16 as feature extraction layers, while preserving only three max-pooling layers. This design choice results in the feature map being reduced to half its original size at each max-pooling layer, yielding an overall reduction factor of 8. Subsequently, multiple DCMs are stacked following the backbone network. Finally, a fully connected layer is incorporated at the network’s backend to reduce the number of channels to 1, thereby generating the predicted density map. The density map is then upsampled to match the original image’s dimensions. This step is pivotal as it facilitates direct loss calculation with the real density map, thus mitigating counting errors attributed to downsampling. Essentially, the predicted density map shares the same dimensions as the ground truth density map, simplifying direct comparison. For CL-DCNN with one DCM, detailed layer parameters are provided in Table 1.

### 3.1. Dilated Contextual Module

**Figure 3:**
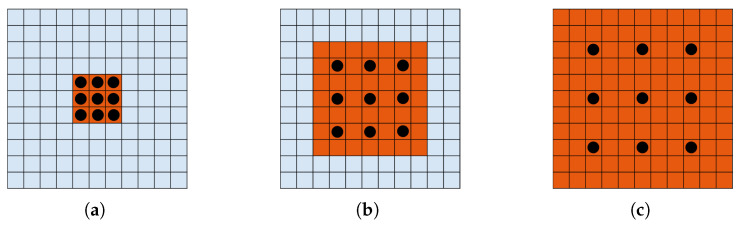
Examples of the receptive field of dilated convolution with different dilation ratio *r*: (**a**) for *r* = 1 with receptive field 3×3 in size, (**b**) for *r* = 2 with receptive field 5×5 in size, and (**c**) for *r* = 3 with receptive field 7×7 in size.

To harness richer contextual information, dilated convolution is integrated into deep layers of convolutional neural networks without compromising the resolution of the feature maps. Specifically, it introduces gaps between input elements, facilitating the capture of a broader context of information. The size of this gap is regulated by a parameter known as the dilation rate. Figure 3 illustrates examples of the receptive field of dilated convolution with a kernel size of 3 × 3 at various dilation rates. Notably, the receptive field remains identical to that of ordinary convolution when the dilation ratio *r* = 1. However, it expands to 5 × 5 and 7 × 7, respectively, as the dilation ratio increases to *r* = 2 and *r* = 3. As the dilation rate escalates, the convolution kernel’s receptive field on the input enlarges, enabling the capture of more contextual information.

Dilated convolution effectively expands the receptive field, enhancing the network’s capacity to discern local information within images. In this study, the 2D dilated convolution is defined as follows:

(1) $$y\left(l,m\right)=\sum_{i=1}^{L}\sum_{j=1}^{M}x\left(l+r\times i,m+r\times j\right)w\left(i,j\right)$$

where *x* and *y* are the input and output of the dilated convolution, respectively, (l,m) is the coordinate of a point on the output *y*, and *w* is the dilated convolution kernel with length *L*, width *M*, and dilation ratio *r*.

To tackle the challenge of diminishing spatial information as the receptive field expands with continuous dilated convolution, we introduce a Dilated Contextual Module (DCM) (depicted in Figure 2). Specifically, the abbreviation DBR denotes the sequence of operations: Dilated Convolution-Batch Normalization-ReLU, while CBR represents Convolution-Batch Normalization-ReLU. The numbers following CBR and DBR denote the kernel size-channel-padding-stride and kernel size-channel-padding-stride-dilation rate, respectively.

As depicted in Figure 2, the proposed DCM comprises two branches. The upper branch of the DCM utilizes a 1 × 1 convolution, effectively reducing the number of channels while retaining contextual information. Meanwhile, the lower branch consists of three dilated convolutions, which expand the receptive field and capture multi-scale information. Assuming the input feature map of the DCM is denoted as *F*, its output can be represented in the following form:

(2) $$DCM\left(F\right)=C(f^{1\times 1}\left(F\right),f^{1\times 1}\left(d_{3}^{3\times 3}\left(f^{1\times 1}\left(F\right)\right)\right))$$

where $f^{1\times 1}$ represents the convolution operation with kernel size 1 × 1, $d_{3}^{3\times 3}$ represents the dilated convolution operation with three convolutional kernels of size 3 × 3, and C(·) represents the channel-wise concatenation.

By integrating these two branches, the DCM effectively balances the trade-off between receptive field size and feature resolution. The 1 × 1 convolution in the upper branch serves as a bottleneck, compressing the input feature maps and reducing the computational cost of subsequent dilated convolutions. This ensures that the model can capture both local and global features without compromising spatial resolution. The cross-layer connections, established by concatenating the output feature maps of both branches, facilitate information flow between deep and shallow features, enhancing the model’s ability to extract informative features from crowd images. Furthermore, these connections not only enable the DCM to effectively capture multi-scale information and improve the network’s ability to discern local details and global context, but also enhance information flow between different layers by concatenating the channel dimensions of both branches. This results in improved performance of the CNN backbone. Finally, the output feature map of the DCM preserves the size and number of channels of the input feature map, rendering it a plug-and-play module that seamlessly integrates into the CNN backbone.

### 3.2. Density Map Generation

In this section, we adopt the methodology established in [2,15] for generating ground-truth density maps of crowd images. Specifically, the presence of a human head target at pixel $x_{i}$ in a crowd image is denoted by the function $\delta (x-x_{i})$. To construct a density map, we begin by creating an all-zero matrix of the same dimensions as the crowd image and set the value at the corresponding pixel to 1. If there are *N* head marks in the crowd images, the resulting image can be generated as follows:

(3) $$H\left(x\right)=\sum_{i=1}^{N}\delta \left(x-x_{i}\right).$$

Applying a geometry-adaptive Gaussian kernel with standard deviation σ to each $\delta (x-x_{i})$, the final density map D(x) can be generated by:

(4) $$D\left(x\right)=H\left(x\right)\cdot G_{\sigma }\left(x\right)=\sum_{i=1}^{N}\delta \left(x-x_{i}\right)\cdot G_{\sigma_{i}}\left(x\right)$$

where · represents the convolution operation and $G_{\sigma_{i}}\left(x\right)$ is a 2D Gaussian kernel centered at $x_{i}$ with a standard deviation of σ:

(5) $$G_{\sigma_{i}}\left(x\right)=\frac{1}{2\pi \sigma_{i}^{2}}e^{-x^{2}/2\sigma_{i}^{2}}$$

where $\sigma_{i}=\beta \cdot \bar{d_{i}}$ with a constant parameter β. $\bar{d_{i}}$ is defined as the average *k* nearest neighbors Euclidean distance:

(6) $${\bar{d}}^{i}=\frac{1}{k}\sum_{j=1}^{k}d_{j}^{i}$$

where $d_{j}^{i}$ represents the distance between the head at pixel $x_{i}$ and the head at pixel $x_{j}$.

The parameter settings of the Gaussian kernel vary across different datasets, contingent upon the sparsity of the crowd distribution. Typically, a fixed standard deviation of σ=15 is employed for relatively uniform crowd distributions. However, in congested datasets where heads appear distorted due to significant angle distortion, the standard deviation is determined as the average distance between the *k* nearest neighbors, with k=3 commonly chosen. Using the density map, the exact number of persons can be inferred by integrating over it, followed by a rounding operation.

### 3.3. Loss Function

As crowd counting is regarded as a pixel-level regression task, the Euclidean distance is commonly employed to assess the similarity between the predicted density map and the ground truth density map. Consequently, the loss function in this study is defined as follows:

(7) $$L\left(\Theta \right)=\frac{1}{2N}\sum_{i=1}^{N}{∥D\left(X_{i};\Theta \right)-D_{i}∥}_{2}^{2}$$

where *N* represents the number of training images, $D(X_{i};\Theta )$ denotes the predicted density of the ith input image $X_{i}$ with a set of learnable parameter Θ, encompassing the weights and biases of the backbone network and DCMs in the proposed CL-DCNN model, and $D_{i}$ stands for the true density map of the training sample $X_{i}$. Additionally, ${∥\cdot ∥}_{2}^{2}$ calculates the squared Euclidean distance between the predicted density map and the true density map for similarity computation.

## 4. Experiments

In the experiments, the proposed method is compared with several representative crowd counting methods on five public datasets. Firstly, detailed information about the datasets and the experimental settings is provided. Subsequently, a comparison evaluation of counting performance between the proposed approach and other state-of-the-art methods reported in the literature is listed and analyzed. Finally, in the last part of the experiment, discussions are conducted to further evaluate the effectiveness of the proposed approach. This includes ablation experiments and comparisons with other typical contextual structures.

### 4.1. Datasets

**Figure 4:**
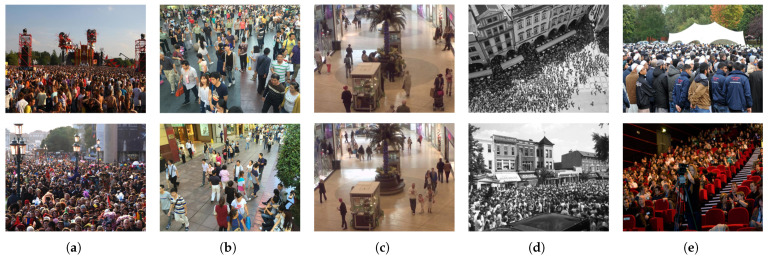
Sample images from benchmark crowd counting datasets: (**a**) ShanghaiTech part A; (**b**) ShanghaiTech part B; (**c**) MALL; (**d**) UCF_CC_50; (**e**) UCF-QNRF.

In this study, experiments are conducted on five publicly available benchmark datasets: ShanghaiTech part A, ShanghaiTech part B, Mall, UCF_CC_50, and UCF−QNRF. Table 2 presents the specific statistics of these datasets. Additionally, representative samples from these datasets are illustrated in Figure 4.

- ShanghaiTech [2] stands as one of the most extensively utilized large-scale crowd counting datasets. It comprises 1198 images and 330,165 annotated head centers. The dataset is divided into two parts, part A and part B, reflecting distinct crowd density distributions. Part A consists of images randomly sourced from the Internet, whereas Part B comprises images captured from a bustling street in the metropolis of Shanghai.
- Mall [16] is a dataset compiled from surveillance videos of shopping centers. The video sequence encompasses 2000 frames and includes a total of 62,325 pedestrians. This dataset encapsulates a broad spectrum of density variations under various lighting conditions, along with instances of severe occlusion between individuals.
- UCF_CC_50 [17], developed at the University of Central Florida, comprises only 50 images with 63,075 annotated head centers. This dataset encompasses a diverse array of scenes, including concerts, protests, stadiums, and marathons, with densities ranging from 94 to 4543. The images in UCF_CC_50 exhibit different viewing angles, resulting in varying degrees of perspective distortion.
- UCF-QNRF [18] stands as a highly challenging dataset, comprising 1535 high-resolution crowd images with a total of 1,251,642 annotated head centers. These images encapsulate a wide spectrum of crowd densities and are captured by surveillance cameras with varying viewpoints and angles. Notably congested, the dataset exhibits head counts ranging from 49 to 12,865.

### 4.2. Implementation Details

To evaluate the performance of crowd counting, two metrics, i.e., mean absolute error (MAE) and root mean square error (RMSE), are widely utilized for comparison. Specifically, MAE measures the mean absolute error between the predicted and true values, while RMSE measures the square root of the mean squared error between the predicted and true values. They are defined as follows:

(8) MAE=1N∑i=1N∥C(Ii)−C^(Ii))∥

(9) RMSE=1N∑i=1N∥C(Ii)−C^(Ii)∥2

where *N* is the number of images in the testing set, $I_{i}$ represents the density map of $X_{i}$, C^(Ii) is the real count of $X_{i}$, $C\left(I_{i}\right)=\sum_{w=1}^{W}\sum_{h=1}^{H}I_{i}^{w,h}$ means the estimated count of $X_{i}$, and *W* and *H* are dimensions of density map, while $I_{i}^{w,h}$ is the pixel at (h,w) of $I_{i}$. The smaller values of MAE and RMSE indicate better performance of the crowd counting model.

Furthermore, all experiments are conducted on a single PC equipped with an Intel(R) Core(TM) i7-8700K CPU and an NVIDIA RTX 2080Ti GPU using PyTorch. Adhering to the general settings found in many literature sources, the original image is randomly cropped to dimensions of 576 × 768 pixels during model training. The number of DCMs for different datasets is set as follows: 1 for ShanghaiTech part A, 3 for ShanghaiTech part B, and 2 for Mall, UCF_CC_50, and UCF-QNRF datasets. Simultaneously, the density map is trimmed to match the image’s size. To augment the training data, random horizontal flipping is applied. The Adam optimizer with a small learning rate of 1 × 10^−5^ is utilized for model training. To facilitate convergence, the density map is multiplied by a factor of 100, a practice adopted across all proposed experiments. The entire network undergoes training for 1000 epochs, with a lower learning rate employed in the final stages to fine-tune the model.

### 4.3. Comparisons with State-of-the-Arts

To demonstrate the effectiveness of the proposed approach, we conduct comparisons with state-of-the-art methods recently published across five datasets. In the following subsections, we present the details of these experiments. In instances where results are not provided by the authors in the corresponding papers, we denote this with a hyphen ‘-’.

#### 4.3.1. Results on the ShanghaiTech Dataset

We compare CL-DCNN with other state-of-the-art methods recently reported on the ShanghaiTech dataset, including part A and part B. The detailed results for each method are listed in Table 3. It is evident that the proposed CL-DCNN achieves comparable performance to other approaches with this dataset. On the one hand, the proposed CL-DCNN demonstrates good performance on the ShanghaiTech part A dataset, with the best MAE value of 52.6 compared to multi-scale feature-based approaches, such as Lw-Count, SCNet, and SASNet. On the other hand, when compared with attention-based methods, such as HA-CNN [19] and CG-DRCN-CC [20], CL-DCNN achieves competitive performance on both the densely populated ShanghaiTech part A dataset and the sparsely populated ShanghaiTech part B dataset, with an MAE/RMSE of 8.1/12.8. From the results in Table 3, it can be concluded that CL-DCNN achieves superior performance compared to the state-of-the-art methods on the part A dataset, indicating that our model effectively captures spatial contextual information and accurately predicts crowd density on this dataset. However, our approach falls slightly behind the state-of-the-art approaches on the part B dataset. This phenomenon may be attributed to the higher level of difficulty in the part B dataset, which includes more complex crowd density distributions and backgrounds. This complexity may lead to CL-DCNN encountering difficulties in accurately estimating crowd density in complex scenes, while the state-of-the-art methods exhibit better generalization ability in this regard.

**Figure 5:**
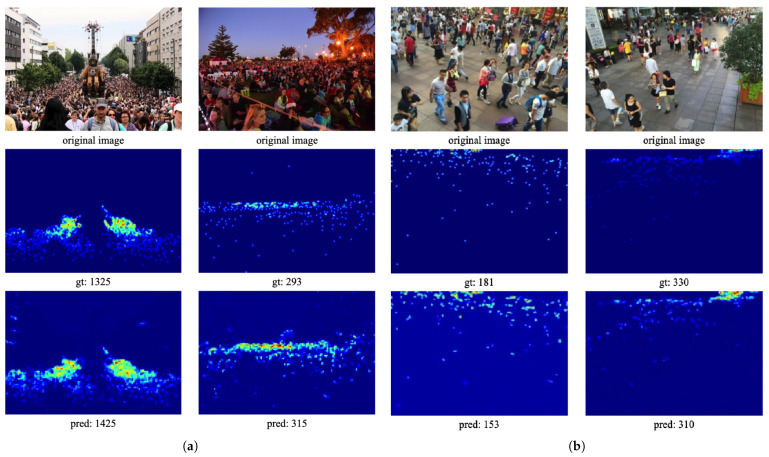
Results on the ShanghaiTech dataset. (**a**) for ShanghaiTech part A and (**b**) for ShanghaiTech part B. The first row shows different crowd test samples. The second row illustrates the ground truth, while the third rows displays the estimated results.

In comparison to state-of-the-art methods, CL-DCNN exhibits a simpler structure yet achieves comparable performance on the Part_B dataset, showcasing the superiority of our approach. These results affirm the effectiveness of CL-DCNN on the ShanghaiTech dataset. Figure 5 illustrates examples of density map prediction results generated by CL-DCNN on the ShanghaiTech dataset. It is evident that the crowd counting results predicted by CL-DCNN are highly accurate and closely approximate the ground truth. Furthermore, these examples underscore the effectiveness of our proposed method in handling complex crowd scenes with varying crowd densities and backgrounds.

#### 4.3.2. Results on the Mall Dataset

**Figure 6:**
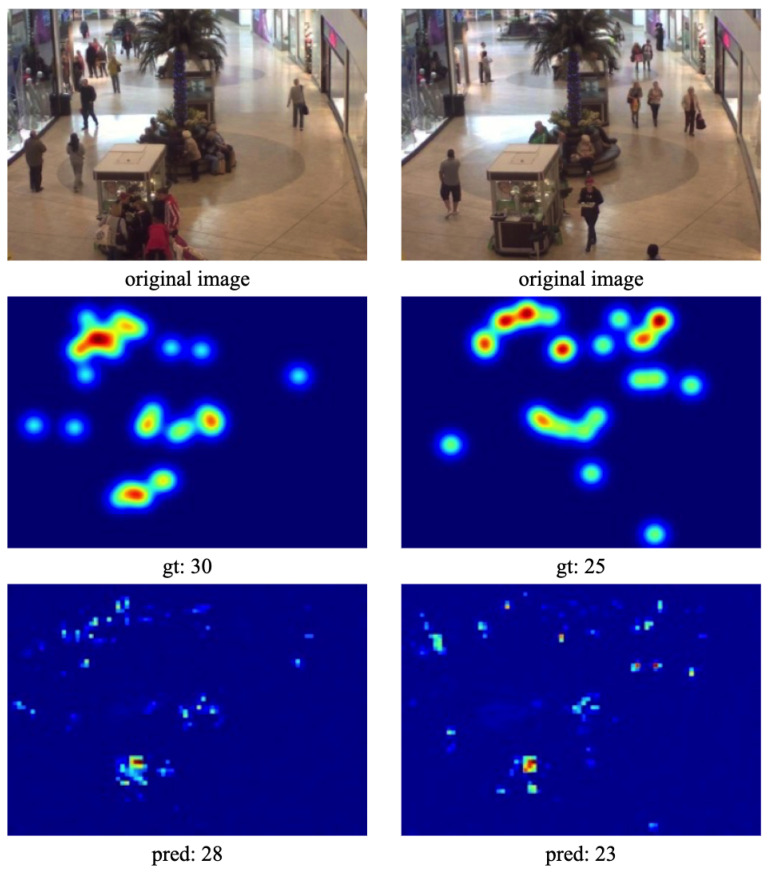
Results on Mall dataset. The first row illustrates different congested test samples, the second row presents corresponding ground truth, and the third row shows estimated results.

For the Mall dataset, the proposed CL-DCNN was compared with 12 other state-of-the-art approaches. Detailed results of all these approaches are listed in Table 4. Among these methods, both CountForest and MCNN employ a multi-column structure, with CountForest constructing a hybrid model for crowd counting by integrating a multi-branch convolutional neural network and deep regression forest. Comparatively, the proposed CL-DCNN achieves better counting performance when contrasted with multi-scale feature-based approaches such as MGF, MLSTN, and SAC-Crowd. Results in Table 4 demonstrate that CL-DCNN achieves the best MAE/RMSE results of 1.55/2.01 on the Mall dataset compared to all these methods. This indicates that CL-DCNN performs admirably in scenes with sparse crowds and significant occlusions. Examples of generated results can be seen in Figure 6.

#### 4.3.3. Results on the UCF_CC_50 and UCF-QNRF Datasets

**Figure 7:**
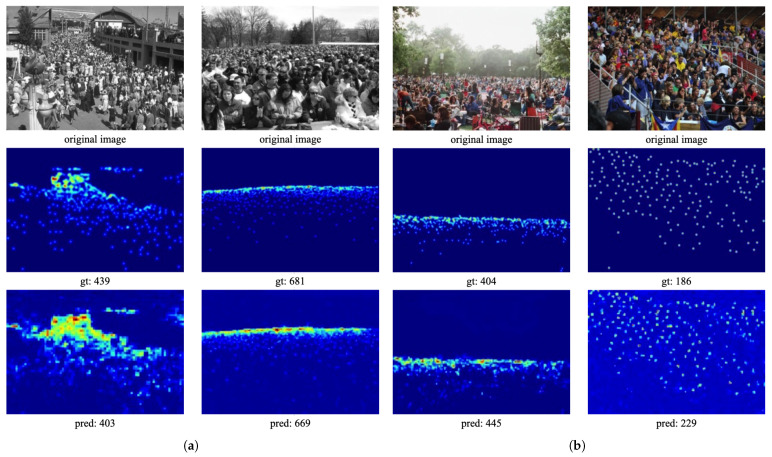
Results on the UCF_CC_50 and the UCF-QNRF datasets (shown in (**a**) and (**b**), respectively). The first row illustrates different congested test samples, the second row presents corresponding ground truth, and the third row shows estimated results. (**a**) UCF_CC_50; (**b**) UCF-QNRF.

For the UCF_CC_50 and UCF-QNRF datasets, we compare the proposed CL-DCNN with other state-of-the-art approaches recently reported, as listed in Table 5. It is evident that CL-DCNN achieves competitive MAE/RMSE values of 181.8/240.6 on the UCF_CC_50 dataset and 96.4/168.7 on the UCF-QNRF dataset. Compared to the other approaches in Table 5, CL-DCNN still demonstrates significant advantages. Selected samples from these two datasets are depicted in Figure 7. It can be inferred that UCF-QNRF poses a greater challenge than UCF_CC_50, featuring a wider range of densities and more noise, making it more difficult to handle. While the cross-layer connections and dilated convolutions utilized in CL-DCNN may not be entirely sufficient to effectively address the complex scenarios in the UCF-QNRF dataset, it still outperforms most state-of-the-art methods, demonstrating its superiority. These results underscore the effectiveness and robustness of our CL-DCNN method for counting and density estimation tasks in crowded scenes. The superior performance of our method on the UCF_CC_50 dataset can be attributed to its ability to handle complex scenes with significant variations in scale and density. Additionally, our method’s competitive performance on the UCF-QNRF dataset showcases its potential for deployment in real-world scenarios, where accurate crowd counting and density estimation are crucial for ensuring public safety and security. Overall, our CL-DCNN method shows promise as a reliable and efficient tool for crowd analysis in various settings.

### 4.4. Ablation Study

In this section, several ablation studies are conducted to assess the effects of different modules in our proposed CL-DCNN method. These studies are carried out on the ShanghaiTech datasets, encompassing both part A and part B.

#### 4.4.1. Number of DCMs

To assess the effectiveness of the proposed DCM, an ablation study was systematically conducted on the ShanghaiTech dataset. The number of DCMs was varied from 0 to 4 in this experiment, and estimation errors measured through MAE and RMSE for part A and part B are presented in Table 6.

The results show noticeable improvements in the proposed approach by adjusting the number of DCMs. Particularly, incorporating one DCM resulted in the best performance, achieving a 17.48% improvement in MAE and a 12.53% improvement in RMSE compared to the baseline approach without DCM for part A. For part B, it achieved 26.1% and 15.93% improvements in MAE and RMSE, respectively. However, increasing the number of DCMs to two resulted in a decrease in performance, possibly due to overfitting and increased model complexity. These findings underscore the importance of carefully selecting the number of DCMs to balance model complexity and performance. Overall, the results demonstrate that the proposed DCM can effectively capture contextual information and enhance the performance of crowd counting methods.

#### 4.4.2. Backbone

In addition to varying the number of DCMs, different backbone networks are employed to evaluate the performance of the proposed approach. Three typical structures, including AlexNet, VGG16, and ResNet50, are utilized in this experiment for the ShanghaiTech dataset. Estimation errors, measured through MAE and RMSE for part A and part B, are listed in Table 7. Specifically, for AlexNet-based CL-DCNN, the first five convolutional layers with two max-pooling layers in AlexNet are utilized as the feature extractor. Then, for VGG16-based CL-DCNN, the first 10 convolutional layers with 3 max-pooling layers in VGG16 are employed to extract features from crowd images. Lastly, for ResNet50-based CL-DCNN, the entire structure of ResNet50 is utilized as the feature extractor without the last fully connected layer. Furthermore, to reduce training costs, all these backbone networks are pre-trained on ImageNet, and we fine-tune them in this experiment.

It can be observed that estimation error varies with different backbone networks. Compared with VGG16 and ResNet50, the MAE and RMSE values of AlexNet-based CL-DCNN for the ShanghaiTech dataset are the highest among these three approaches. This is because the structure of AlexNet is much simpler than the other two, which may lead to certain underfitting for the testing dataset. However, the parameter spacing of ResNet50 is relatively larger than the other two backbone networks, which may result in overfitting to some extent. Consequently, the estimation error in MAE and RMSE is inferior to VGG16-based CL-DCNN for the ShanghaiTech dataset. In addition, the CPU time for fine-tuning also increases with the parameter space of different backbone networks. Generally, the performance of VGG16-based CL-DCNN is the best among these three methods because of its comparatively situated parameter space for the size of the ShanghaiTech dataset.

### 4.5. Discussions

In this section, several experiments are conducted to discuss the proposed approach, including comparisons with other contextual models, computational complexity analysis, and the quality of the estimation results.

**Figure 8:**
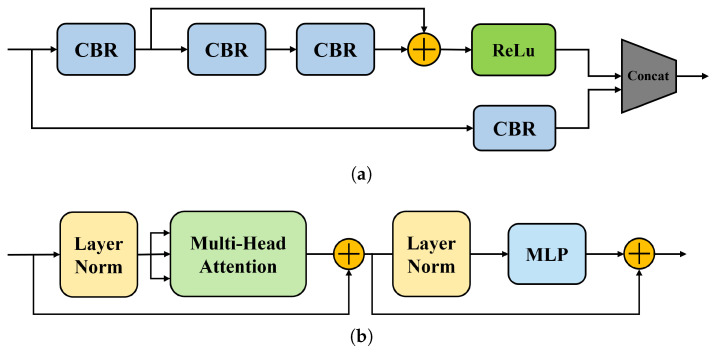
Other contextual structures for performance comparison. (**a**) CSP network; (**b**) Transformer learning.

Firstly, the core idea of DCM is established with dilated convolution, which concatenates contextual features and reduces the loss of detailed information. It is worth noting that there are other structures, such as the Cross-Stage Partial (CSP) network [52] and transformer learning [53], that can achieve similar effects, as listed in Figure 8a,b, respectively.

The CSP network is similar to DCM in that it divides shallow feature maps into two parts in the channel dimension. One part is propagated forward through the feature extraction module, while the other part is merged with the output through the cross-stage hierarchy structure, resulting in richer gradient combinations. Unlike DCM, the CSP network uses residual blocks for feature extraction. The outputs of the two parts are merged together to achieve a richer gradient combination. On the other hand, transformer learning employs a self-attention mechanism to connect contextual features. To verify the effectiveness of the proposed approach, these approaches are implemented on a VGG16 backbone network and evaluated on parts A and B of the ShanghaiTech dataset. The comparative results of these approaches are presented in Table 8.

Experimental results in Table 8 indicate that the proposed CL-DCNN model outperforms other methods in terms of crowd counting, demonstrating the effectiveness of the DCM in capturing multi-scale information. Specifically, our method achieved a 25.8% improvement in terms of MAE and a 22.55% improvement in terms of RMSE for the CSP network, and a 62.5% improvement in terms of MAE and a 57.9% improvement in terms of RMSE for the transformer learning. For the CSP network, it aims to capture the global and local contextual information by fusing features with different scales. One possible reason for the relatively poor performance of transformer learning is that it may not be well-suited for capturing local spatial information, which is important for accurate crowd counting. Transformer learning relies heavily on self-attention mechanisms, which are designed to capture long-range dependencies between different parts of an input sequence. However, in crowd counting, the local spatial arrangement of people is often more important than long-range dependencies. The results of the experiments demonstrate the effectiveness of CL-DCNN in capturing both local and global contextual information using dilated convolution operations. Overall, the proposed CL-DCNN method outperforms state-of-the-art methods in crowd counting. The DCM proves to be a useful addition to enhance the performance of CNN-based crowd counting methods.

Secondly, to reduce the complexity of the CL-DCNN, the VGG16 network is used as a backbone for feature extraction. Table 9 includes information on the computation complexity in terms of the number of parameters and runtime. Despite the Cascaded-MTL [21] presenting the lightest model with only 0.12M parameters and the fastest runtime among these methods, it has the worst estimation performance, which may be caused by the underfitting of the model. During the evaluation phase, the proposed CL-DCNN takes almost 11M parameters to process a frame with 576 × 768 pixels for all testing datasets on an RTX 2080ti GPU. With a pre-trained model on ImageNet, the CL-DCNN provides a faster execution time than the Switching-CNN [22] and CP-CNN [23] models. Moreover, for PCC Net [25], its parameter space is as light as the Cascaded-MTL model, but its runtime is even higher due to the inferior running platform. As shown in Table 9, the parameter number of the proposed CL-DCNN is one-third of the U-ASD network [12] and with a lower runtime. After considering the running platform and parameter quantity comprehensively, the effectiveness of the proposed CL-DCNN and U-ASD network is equivalent.

**Figure 9:**
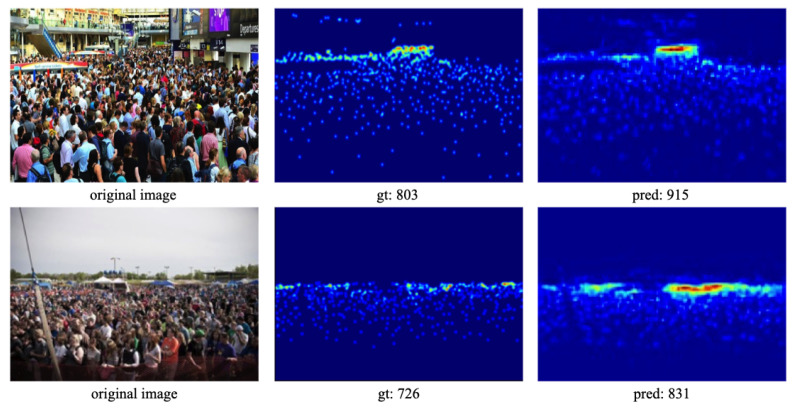
Some poor samples of the proposed CL-DCNN for the ShanghaiTech dataset.

Finally, due to perspective distortion and background noise, the performance of the proposed CL-DCNN is significantly degraded in some crowd images. Specifically, a few sample images from the ShanghaiTech dataset are shown in Figure 9. The image in the first row is affected by a cluttered background, while the one in the second row is notably distorted by perspective issues. These drastic scale changes resulting from these factors pose significant challenges for crowd counting.

## 5. Conclusions

In this study, we introduce CL-DCNN, a dilated convolutional neural network that integrates a dilated contextual module into its architecture to capture both global and local contextual information. Our experimental findings demonstrate that CL-DCNN surpasses state-of-the-art methods across five publicly available datasets. Moreover, despite its advanced performance, CL-DCNN maintains a reasonable computational overhead compared to alternative approaches. Nonetheless, there are still inherent limitations in our proposed method. Future research should explore the integration of lighter-weight CNN architectures with the dilated contextual module to enhance its applicability in practical scenarios, such as unmanned aerial vehicle platforms.

## Figures and Tables

**Table 1:** Structures and detailed parameter settings of the proposed CL-DCNN.

Structures	Layer Types	Number of Channels	Kernel Size	Padding Size	Stride	Dilation Rate
Backbone	conv	64	3 × 3	1	1	1
	conv					
	max-pooling	-	2 × 2	-	2	-
	conv	128	3 × 3	1	1	1
	conv					
	max-pooling	-	2 × 2	-	2	-
	conv	256	3 × 3	1	1	1
	conv					
	conv					
	max-pooling	-	2 × 2	-	2	-
	conv	512	3 × 3	1	1	1
	conv					
	conv					
DCM_i (1 ≤ i ≤ m)	Branch_1	256	1 × 1	0	1	1
		128	1 × 1	0	1	1
		64	3 × 3	2	1	2
	Branch_2	32	3 × 3	2	1	2
		16	3 × 3	2	1	2
		256	1 × 1	0	1	1
fully connected layer		1	1 × 1	0	1	1

**Table 2:** Statistics of the benchmark datasets used in the experiments. Total, Min, Avg, and Max represent the total number, the minimum, average number, and maximum number of instances in the datasets, respectively.

Dataset	Number of Images	Training/Testing	Average Resolution	Statistics			
				**Total**	**AVG**	**Min**	**Max**
ShanghaiTech part A	482	300/182	589 × 868	241,667	501	33	3139
ShanghaiTech part B	716	400/316	768 × 1024	88,488	123	9	578
Mall	2000	800/1200	320 × 240	62,325	31	13	53
UCF_CC_50	50	-	2101 × 2888	63,974	1280	94	4543
UCF-QNRF	1535	1201/334	2013 × 2902	1,251,642	815	49	12,865

**Table 3:** Comparison of the proposed approach with the other state-of-the-art crowd counting approaches on the ShanghaiTech dataset (part A and part B).

Methods	Part A		Part B	
	**MAE**	**RMSE**	**MAE**	**RMSE**
Cascaded-MTL [21] (2017)	101.3	152.4	20.0	31.1
Switching-CNN [22] (2017)	90.4	135.0	21.6	33.4
CP-CNN [23] (2017)	73.6	106.4	20.1	30.1
CSRNet [9] (2018)	68.2	106.4	10.6	16.0
HA-CCN [19] (2019)	62.9	94.9	8.1	13.4
S-DCNet [24] (2019)	58.3	95.0	6.7	10.7
ACSPNet [10] (2019)	85.2	137.1	15.4	23.1
PCC Net [25] (2020)	73.5	124	11.0	19.0
CG-DRCN-CC [20] (2020)	60.2	94	8.5	14.4
LSC-CNN [26] (2020)	66.4	117.0	8.1	12.7
ADSCNet [27] (2020)	55.40	97.7	6.4	11.3
SCNet [28] (2021)	58.5	99.1	8.5	13.4
Density CNN [29] (2021)	63.1	106.3	9.1	16.3
EPA [30] (2021)	60.9	91.6	7.9	11.6
S3 [31] (2021)	57	96	**6.3**	**10.6**
DFN [32] (2021)	77.58	129.7	14.1	21.10
U-ASD Net [12] (2021)	64.6	106.1	7.5	12.4
ChfL [33] (2022)	57.5	94.3	6.9	11
Lw-Count [7] (2022)	69.7	100.5	10.1	12.4
PFDNet [11] (2022)	53.8	**89.2**	6.5	10.7
CL-DCNN (ours)	**52.6**	90.9	8.1	12.8

**Table 4:** Comparison of the proposed approach with the other state-of-the-art crowd counting approaches on the Mall dataset.

Methods	MAE	RMSE
Ridge Regression [34] (2012)	3.59	19.0
MCNN [2] (2016)	2.24	8.5
DE-VOC [35] (2016)	2.7	2.1
Bidirectional ConvLSTM [36] (2017)	2.10	7.6
weighted-VLAD [37] (2018)	2.4	9.1
DRSAN [38] (2018)	1.72	2.10
SAC-Crowd [39] (2019)	2.3	3.0
MGF [40] (2019)	1.89	7.29
ACSPNet [10] (2019)	1.76	2.24
LSTN [41] (2019)	2.00	2.50
ST-CNN [42] (2019)	4.03	5.87
CountForest [4] (2020)	2.25	6.21
MLSTN [43] (2020)	1.80	2.42
TAN [44] (2020)	2.03	2.60
FSC [45] (2020)	3.71	4.66
U-ASD Net [12] (2021)	1.8	2.2
GRGAF-ST [46] (2022)	1.61	2.07
CL-DCNN (ours)	**1.55**	**2.01**

**Table 5:** Comparison of the proposed approach with the other state-of-the-art crowd counting approaches on the UCF_CC_50 and UCF-QNRF dataset.

Methods	UCF_CC_50		UCF-QNRF	
	**MAE**	**RMSE**	**MAE**	**RMSE**
Cascaded-MTL [21] (2017)	322.8	341.4	251.85	513.92
CP-CNN [23] (2017)	295.8	320.9	–	–
Switching-CNN [22] (2017)	318.1	439.2	227.71	444.78
CSRNet [9] (2018)	266.1	397.5	120.3	208.5
CA-Net [3] (2019)	–	–	107	183
HA-CCN [19] (2019)	256.2	348.4	118.1	180.4
S-DCNet [24] (2019)	204.2	301.3	104.4	176.1
SFCN [47] (2019)	214.2	318.3	102.0	171.4
SD-CNN [48] (2019)	235.74	345.6	–	–
LSC-CNN [26] (2020)	225.6	302.7	120.5	218.2
PCC Net [25] (2020)	240.0	315.5	246.41	247.12
EPA [30] (2021)	250.1	342.1	–	–
Density CNN [29] (2021)	244.6	341.8	101.5	186.9
SRNet [13] (2021)	184.1	**232.7**	108.2	177.5
DFN [32] (2021)	402.3	434.1	218.2	357.4
SS-CNN [49] (2021)	229.4	325.6	115.2	175.7
SDS-CNN [50] (2021)	–	–	112	173
U-ASD Net [12] (2021)	232.3	**217.8**	–	–
KDMG [51] (2022)	–	–	99.5	173.0
PFDNet [11] (2022)	205.8	289.3	–	–
Lw-Count [7] (2022)	239.3	307.6	149.7	238.4
CL-DCNN (ours)	**181.8**	240.6	**96.4**	**168.7**

**Table 6:** Estimation error on the ShanghaiTech dataset w.r.t. different number of DCM.

Number of DCMs	Part A		Part B	
	**MAE**	**RMSE**	**MAE**	**RMSE**
0	63.82	104.02	10.97	14.84
1	**52.66**	**90.98**	10.06	13.16
2	57.64	96.32	8.92	13.35
3	60.83	100.12	**8.1**	**12.8**
4	62.32	102.47	9.15	12.94

**Table 7:** Estimation error on the ShanghaiTech dataset with respect to different settings of backbone network.

Backbone	Part A		Part B	
	**MAE**	**RMSE**	**MAE**	**RMSE**
AlexNet	56.40	96.72	9.45	13.48
VGG16	**52.66**	**90.98**	**8.1**	**12.8**
ResNet50	54.52	95.21	9.94	14.07

**Table 8:** Comparison of different contextual structures on part A and part B of the ShanghaiTech dataset.

Models	Part A		Part B	
	**MAE**	**RMSE**	**MAE**	**RMSE**
CSP Network [52] (2020)	71.04	117.47	11.7	15.3
Transformer Learning [53] (2022)	66.1	105.1	9.3	14.1
CL-DCNN (Ours)	52.66	90.98	8.1	12.8

**Table 9:** Computational complexity of the proposed CL-DCNN and other state-of-the-art approaches.

Methods	Parameters	Runtimes (ms)	Device	Pre-Train
Cascaded-MTL [21]	**0.12M **	**3**	TITAN-X	–
Switching-CNN [22]	15.1M	153	–	*√*
CP-CNN [23]	62.9M	5113	–	*√*
PCC Net [25]	0.55M	89	1080Ti	–
U-ASD Net [12]	31.4M	94	Tesla V100	*√*
CL-DCNN (Ours)	11M	62	2080Ti	*√*

## Data Availability

The data that support the findings of this study are openly available.
